# Behavioral testing in rodent models of orofacial neuropathic and inflammatory pain

**DOI:** 10.1002/brb3.85

**Published:** 2012-08-15

**Authors:** Agnieszka Krzyzanowska, Carlos Avendaño

**Affiliations:** Department of Anatomy, Histology and Neuroscience, Autonoma University of Madrid, Medical SchoolMadrid, Spain

**Keywords:** facial pain, pain models, TMD, trigeminal neuralgia

## Abstract

Orofacial pain conditions are often very debilitating to the patient and difficult to treat. While clinical interest is high, the proportion of studies performed in the orofacial region in laboratory animals is relatively low, compared with other body regions. This is partly due to difficulties in testing freely moving animals and therefore lack of reliable testing methods. Here we present a comprehensive review of the currently used rodent models of inflammatory and neuropathic pain adapted to the orofacial areas, taking into account the difficulties and drawbacks of the existing approaches. We examine the available testing methods and procedures used for assessing the behavioral responses in the face in both mice and rats and provide a summary of some pharmacological agents used in these paradigms to date. The use of these agents in animal models is also compared with outcomes observed in the clinic.

## Introduction

### Orofacial pain characteristics

Pain in the face and mouth region (orofacial pain) may be particularly distressing to the patient due to the special psychological and emotional context of this body area. The prevalence is high: some reports estimate that around 20% of the population is affected by some sort of orofacial pain (Lipton et al. 1993; Macfarlane et al. 2002). While most of these will be dental, over 5% can be chronic, with higher incidence in older patients (Zakrzewska 2010). It is also possible that some chronic cases are overlooked by the general practitioner or dentist who usually is the first contact for many patients (Kitt et al. 2000; Koopman et al. 2009; Zakrzewska 2009).

Nondental conditions which specifically affect the trigeminal nerve include temporomandibular disorders (TMD), burning mouth syndrome, and, most commonly, trigeminal neuralgia (TN; Kitt et al. 2000; Sessle 2005; Koopman et al. 2009, 2011). Woda and colleagues have proposed a classification of chronic orofacial pain conditions into three broad groups, based on the symptoms present. The pain types were grouped either as (1) “neuralgias” which included TN and posttraumatic neuralgia, (2) “neurovascular and tension type” – including migraines, cluster headache, and tension type headaches, and (3) “persistent idiopathic orofacial pain” (Woda et al. 2005). The last group included stomatodynia (also known as burning mouth syndrome), arthromyalgia (TMD), and atypical facial pain. The first group clearly can be characterized as “neuropathic” pain while in the last group, although most of the disorders (such as TMD) have an inflammatory component, others are more difficult to characterize and may not be strictly “inflammatory” (see below).

In many cases, orofacial pain may be idiopathic (might arise without any obvious trigger or identifiable cause) – such as burning mouth syndrome and atypical facial pain (Zakrzewska 2009) – however, some conditions can result from indentified pathologies, such as herpes (postherpetic neuralgia) or multiple sclerosis (responsible for some cases of TN; Cruccu et al. 2009), as well as trauma to facial structures and cancer (Kitt et al. 2000; Watson 2004).

In general, the symptoms are often severe and disturbing and frequently become not responsive to therapy, sometimes needing invasive surgical intervention (Kitt et al. 2000; Zakrzewska 2009; Koopman et al. 2011). It is clear that there is a need for more effective pharmacological agents.

### Scope of review

This review intends to present a comparative summary of the currently available pain models of the orofacial area in the commonly used laboratory rodents. In the last two decades, research into pain mechanisms has shown considerable progress; however, most of the basic science research in this field has been done in the limbs and trunk, due to possibilities of uncomplicated surgical manipulations and the ease of applying stimuli to sciatic-innervated areas for behavioral observations (Le Bars et al. 2001). Whereas many of the findings from other parts of the body can be applied to the orofacial region, the cellular composition and circuitry of the trigeminal system vary to some extent with respect to their spinal counterparts (Kruger and Young 1981; Bereiter et al. 2000). In contrast to most other main nerve trunks, the first and second trigeminal branches are purely sensory, and the motor (masticatory) component of the third branch is distinctly separated from the sensory component by a thick perineurial sheath, from the trigeminal root to the proximal part of the branch. Moreover, there are several human pain conditions that affect specifically the trigeminal nerves and a differential sensitivity to certain drugs in neuropathic pain in the trigeminal versus other territories has been observed (Idanpaan-Heikkila and Guilbaud 1999; Watson 2004). It is therefore valuable to develop specific animal models for the orofacial area. This review presents an overview of the rodent models developed for the trigeminal area over the last 20 years, with the emphasis on behavioral tests. To date, most reviews have overviewed the mechanistic components of trigeminal pain but none have focused specifically on the various behavioral testing methods available in rodents. For reviews which specifically elucidate the mechanisms of orofacial pain, see for example Sessle (2005), Hargreaves (2011), Takeda et al. (2011), Iwata et al. (2011).

The scope of this review will span basic research on the rodent skin and mucosa, and muscular and articular sensory territories of the trigeminal nerve that has been published in the last few decades. For reasons of simplicity, and because these topics have already been broadly discussed in the literature, models that involve specific target organs such as teeth, the eye, and salivary glands will be omitted. Useful reviews and reports are available on dental pain (Cooper and Desjardins 2010; Tarsa et al. 2010; Hargreaves 2011), eye pain (Tashiro et al. 2010; Marquart 2011), and salivary gland pain (Ogawa et al. 2003). Cancer pain has certain inflammatory components and sometimes some neuropathic pain aspects (Mantyh et al. 2002; Benoliel et al. 2007). The behavioral evaluation methods used in animal models of orofacial cancer pain are similar to those used in other orofacial pain models (Nagamine et al. 2006; Ono et al. 2009; Harano et al. 2010) and this topic will also not be explored in this review. Finally, chronic primary headaches, including migraines, fall into craniofacial disorders but are not usually considered “orofacial” conditions^1^ (Zakrzewska 2009) and thus will not be discussed.

Of particular interest will be models of TN, TMD, and facial muscle pain and other models of cutaneous nociception in rodent orofacial pain. While several recent reviews have summarized some of the techniques used to induce neuropathic or inflammatory pain in the facial region (Khan and Hargreaves 2010; Iwata et al. 2011), none specifically centerd on the types of behavioral evaluation techniques available for testing, nor were the pharmacological tools commonly used in animal models compared with the treatments used in the clinic (this being important, as for an animal model to be applicable for future drug testing, it first needs to be validated with clinically used drugs). This review aims to fill this gap as well as present a wide overview of both inflammatory and neuropathic models currently used in laboratory rodents.

## Pain Models

### Inflammatory pain models

Tissue injury results in the release of various inflammatory agents from the damaged endothelial cells and blood vessels. Many of these inflammatory agents activate primary sensory neurons and attract immune response cells, which in turn can release more inflammatory factors (McMahon et al. 2005, 2006). For a recent review of peripheral and central mechanisms of pain in orofacial inflammation see Sessle (2011).

Most peripheral inflammation models involve injection of an inflammatory agent into the area of interest. The inflammatory agents used in pain models range from irritant chemicals (carrageenan, formalin), microbial cell wall fragments or toxins (lipopolysaccharide [LPS], Complete Freund's Adjuvant [CFA], zymosan), to agents that directly activate specific receptors on primary sensory neurons (capsaicin, mustard oil). Following application of such agents, an inflammatory reaction follows which includes edema, fever, cell migration, erythema, allodynia, and hyperalgesia (Marchand et al. 2005).

The inflammatory models in the sciatic region are widely developed. The ease of subdermal injection into the plantar region of the foot and the anatomy of the sciatic nerve and the lumbar ganglia and spinal cord make it the region of choice for most pain studies. Several testing paradigms have been developed, which involve nociceptive stimulation of the rodent hindpaw with heat (Hot plate, Plantar test) and mechanical stimulation (von Frey, Randall-Selitto; see below).

So far, inflammatory substances such as CFA (Zhou et al. 1999; Imbe et al. 2001; Hanstein et al. 2010; Krzyzanowska et al. 2011; Shinoda et al. 2011), carrageenan (Yeo et al. 2004, 2008; Neubert et al. 2005a; Vahidy et al. 2006; Poh et al. 2009; Tang et al. 2009), capsaicin (Pelissier et al. 2002; Quintans-Junior et al. 2010), and formalin (Clavelou et al. 1989; Luccarini et al. 2006; Borsani et al. 2009; Bornhof et al. 2011) have been most frequently used in the orofacial region of rats and mice (see Table 1). While the two latter substances elicit spontaneous pain which allows for observation of grooming, scratching, and rubbing behaviors in response to the application of the inflammatory agent, CFA has mostly been used in expression and electrophysiology studies and relatively few studies involved behavioral assessment post-CFA application (Imbe et al. 2001; Hanstein et al. 2010; Shinoda et al. 2011). Haas et al. (1992) proposed a model of acute inflammation in the facial region which involved mustard oil application into periarticular temporomandibular tissue of rats. They measured the inflammation by Evans blue extravasation, however, no pain behavior was measured. More recently, Ahn et al. have used subcutaneous interleukin-1β (IL-1β) injections into the vibrissal pad of rats to induce mechanical allodynia in the face and were successful in behaviorally quantifying it with the air puff method (Ahn et al. 2004; Jung et al. 2006a,b; see below). The inflammatory substance tends to be chosen on the type of behavioral testing that will be performed and on the duration of the response (e.g., formalin – short – hours; CFA – long – up to a few days). Mustard oil and capsaicin have the disadvantage of activating only a subset of nociceptive receptors while other substances such as CFA result in an extensive inflammatory response which may not be consistent with features observed clinically. Nevertheless, the use of these substances is established in the studies of inflammatory pain, and the efficacy of some clinically used drugs in abolishing the experimentally induced inflammation validates them as useful in both spinal and trigeminal pain studies (see below).

**Table 1:** Summary of inflammatory models of orofacial pain in rodents. Table shows the different types of orofacial models with an inflammatory component in mice and rats, together with their methodology for induction of the model and behavioral testing. Only studies with behavioral analyses are presented

Animal	Strain	Type of model	Where	Stimulus	Restraint method	References^1^
Rat	Sprague–Dawley	Formalin	Upper lip injection	Spontaneous grooming behavior observed	None	Clavelou et al. (1989)
Rat	Sprague–Dawley	CFA	Intra-TMJ or perioral injection	Von Frey	Animal habituated to stand on its hind paws and lean against the experimenter's hand	Ren and Dubner (1999)
Rat	Sprague–Dawley	CFA	Intra-TMJ	Meal-pattern analysis	None	Harper et al. (2000)
Rat	Sprague–Dawley	CFA	Masseteric injection	Bite force observed	None	Ro (2005)
Rat	Not mentioned	CFA	Intra-TMJ or perioral injection	Thermal (radiant heat source)	Light anesthesia	Imbe et al. (2001)
Rat	Sprague–Dawley	CFA	Intra-TMJ or masseteric injection	Operant behavior paradigm involving measurement of food intake	None	Thut et al. (2007)
Rat	Sprague–Dawley	Capsaicin	Vibrissal pad injection	Spontaneous grooming behavior observed	None	Pelissier et al. (2002)
Rat	Sprague–Dawley	Mustard oil	Intra-TMJ	Spontaneous grooming behavior observed	None	Hartwig et al. (2003)
Rat	Sprague–Dawley	IL-1β	Vibrissal pad (delivered subcutaneously through an implanted catheter)	Air puff	None	Ahn et al. (2004)
Rat	Sprague–Dawley	Carageenan	Mid cheek injection	Heat	Operant behavior paradigm	Neubert et al. (2005a)
Rat	Wistar	Carageenan	Upper lip injection	Cold (tetrafluoroethane spray), grooming behavior observed	None – tested in cage	Chichorro et al. (2006)
Rat	Sprague–Dawley	Menthol	Mid cheek injection	Cold	Operant behavior paradigm	Rossi et al. (2006)
Rat	Sprague–Dawley	Capsaicin cream	Cheek (topical)	Mechanical	Operant behavior paradigm	Nolan et al. (2011)
Mouse	NMRI	Formalin	Upper–lip injection	Spontaneous grooming behavior observed	None	Luccarini et al. (2006)
Mouse	C57BL/6, BALB/c	Carageenan	Maxillary injection	Von Frey	Animals placed in a large box and stimulated from above	Tang et al. (2009); Vahidy et al. (2006); Yeo et al. (2004)
Mouse	C57BL6 versusTRPV1 k/o	TRPV1 k/o		Heat	Operant behavior paradigm	Neubert et al. (2008)
Mouse	Swiss	Capsaicin/glutamate	Vibrissal pad injection	Spontaneous grooming behavior observed	None	Quintans-Junior et al. (2010)
Mouse	FVBN	CFA	Tempo mandibular joint/masseter muscle injection	Gnawing through a foam or plastic dowel	“Dolognawmeter” set up	Dolan et al. (2010)
Mouse	C57BL/6	CFA	Whisker pad	Von Frey and Air puff	Loose box-restraint	Krzyzanowska et al. (2011)
Mouse	Balb/c and C57BL/6	CFA	Submandibular skin	Von Frey	Animal placed on a mesh floor, covered by a mesh cup	Hanstein et al. (2010)
Mouse	C57BL/6	CFA	Upper lip injection	Thermal (radiant heat source)	Restraint in plastic tube after isofluorane sedation	Shinoda et al. (2011)

CFA, Complete Freund's Adjuvant; TMJ, temporomandibular joint; IL-1β, interleukin 1β; TRPV1, transient receptor potential cation channel subfamily V member 1.

1 Only the earliest reference to the model is indicated.

Musculoskeletal disorders can have a broad inflammatory component. A variety of syndromes affecting the temporomandibular joint (TMJ) area, collectively called TMD are a common complaint (Sessle 2005). These include TMJ inflammation (often arthritis-related), joint stiffness or dislocation, and muscle pain (Zakrzewska 2009; Mujakperuo et al. 2010; Benoliel et al. 2011). The majority of TMD models involve injection of CFA or other irritant substances such as mustard oil, formalin, and carrageenan into the TMJ (Bereiter and Benetti 1996; Ren and Dubner 1999; Imbe et al. 2001; Roveroni et al. 2001; Hartwig et al. 2003). Interestingly, some TMJ disorders can lack inflammatory changes, and are associated to neuromuscular dysfunction and muscular pain (Stohler 1999; Lam et al. 2005; Cairns 2010). It is thought that peripherally acting glutamate is involved in sensitizing the nociceptors and thus eliciting pain (Lam et al. 2005; Sessle 2011) Peripheral, intramuscular or intraarticular glutamate injections have been used to study orofacial muscle sensitization in rats (Cairns et al. 2002; Lam et al. 2005; Ro and Capra 2006; Fischer et al. 2008) and glutamate-induced nociception in mice (Quintans-Junior et al. 2010). Glutamate injections are also used for studies of TMD in human subjects (Castrillon et al. 2008). Other orofacial muscle pain models involve the ligature of masseter muscle's tendon (Guo et al. 2010), the injection of CFA into the masseter muscle (Ambalavanar et al. 2006), and the stretching or electrically induced contraction of the masseter muscle (Dessem et al. 2010). Importantly, the intramuscular injection of acidic saline, a manipulation which induces mechanical hyperalgesia in the spinal regions (Sluka et al. 2001) does not result in any marked hyperalgesia in the orofacial region, further highlighting differences between the trigeminal region and the rest of the body (Ambalavanar et al. 2007). Of the above models, the one involving the stretching of the masseter muscle may be the most akin to the human conditions as it involves the natural contractility and movement of muscle and shows a similar pathophysiology (Dessem et al. 2010).

Animals other than rats and mice are rarely used in orofacial inflammatory pain models, however, some studies have been performed in rabbits (TMJ inflammation; Swift et al. 1998; Stoustrup et al. 2009) and guinea pigs (skin inflammation; Neubert et al. 2000).

### Neuropathic pain models

Rats and mice have been the animals of choice wherein most, if not all, neuropathic pain models have been developed. And, in general, rats preceded mice as models where most neuropathy-inducing maneuvers have been tried.

The direct damage to a nerve (cutting, ligating, or crushing) results in prominent changes in the expression of various molecules in the dorsal rot ganglias (DRGs) or trigeminal ganglions (TGs) of the affected nerves, leading to the emergence of neuropathic pain.

The neuropathic pain models involve ligating and cutting a whole nerve or parts of a nerve, or placing several loose ligatures around a nerve. Of the various nerve injury models used in the sciatic region, the most applicable to the facial region has proven to be the chronic constriction injury (CCI) model, which involves tying loose ligatures around the nerve (Vos et al. 1994; Khan and Hargreaves 2010). The infraorbital nerve (IoN; maxillary branch of the trigeminal nerve) branches peripherally in a fan-like fashion distal to the infraorbital foramen, and this is where the surgical manipulations are most easily executed. Due to its branching, a wide ligature is necessary over the entire width of the nerve in order to “bunch up” all the branches of the IoN. The tightness of the ligature is important: too loose produces no pain behavior while too tight produces anesthesia (Martin et al. 2010; Krzyzanowska et al. 2011). Such manipulation of the IoN in rats results in behavioral abnormalities which can be compared with some of the symptoms observed in TN such as mechanical hyperalgesia, air-puff allodynia, and paraesthesias/dysaesthesias (Vos et al. 1994, and personal observations). An alternative way of accessing the IoN is from inside of the mouth (Imamura et al. 1997). The advantage of this last approach would be the avoidance of a skin incision and thus sensitization of the “testing area,” but it is likely to hamper feeding. This and the relative difficulty of surgery in this model are probably responsible for its not having been more generally adapted.

Other neuropathic models in the facial region involve transecting or crushing the IoN or one of the other sub-branches of the trigeminal: inferior alveolar, mental, or lingual nerves (all of them branches of the mandibular nerve; Nomura et al. 2002; Iwata et al. 2004; Seino et al. 2009). The choice of different nerves may be based on the relative ease of surgery (easy access, the nerve does not fan-out like the IoN) and can also depend on the proposed evoked behavior stimulation area (e.g., from below). The nerve can also be damaged with the aid of photo-irradiation with an argon ion laser (Eriksson et al. 2005). An alternative manipulation, which also results in neuropathic pain is the compression of the TG (or its root) and subsequent local demyelination, features that epitomize the causes of TN (Kitt et al. 2000; Devor et al. 2002). Other authors have developed a series of models which involve such trigeminal compression or demyelination with the aid of agar (Ahn et al. 2009b) or the demyelinating agent, lysophosphatidic acid (LPA; Ahn et al. 2009a). Examples of neuropathic orofacial models are summarized in Table 2.

**Table 2:** Summary of neuropathic models of orofacial pain in rodents. Table shows the different types of neuropathic pain orofacial models in mice and rats, together with the methodology followed for induction of the model and behavioral testing. Only studies with behavioral analyses are presented

Animal	Strain	Type of model	Where	Stimulus	Restraint method	References^1^
Rat	Sprague–Dawley	CCI	IoN	Von Frey	No restraint	Vos et al. (1994)
Rat	Sprague–Dawley	CCI	IoN	Thermal (radiant heat source)	Box restraint	Imamura et al. (1997)
Rat	Sprague–Dawley	CCI	IoN	Von Frey; spontaneous eye blinking observed; mechanical stimulus in the operant behavior paradigm	None for von Frey and spontaneous behavior; operant behavior paradigm cage	Vit et al. (2008)
Rat	Wistar	CCI	IoN	Cold (tetrafluoroethane spray)	None – tested in cage	Chichorro et al. (2006)
Rat	Wistar	CCI	IoN	Thermal (radiant heat source)	Animal hand held	Chichorro et al. (2009)
Rat	Sprague–Dawley	Photochemical irradiation of nerve	IoN	Thermal (radiant heat source)/Von Frey	Animal “gently held”	Eriksson et al. (2005)
Rat	Sprague–Dawley	Transection	Inferior alveolar nerve	Von Frey	Animals trained to poke nose through a hole and drink. Stimulation when nose poking out	Nomura et al. (2002)
Rat	Sprague–Dawley	TG compression	Trigeminal ganglion	Air puff and pin prick. Spontaneous scratching behavior quantified	None – tested in cage	Ahn et al. (2009b)
Rat	Sprague–Dawley	Injection of LPA in to TG (demyelination)	Trigeminal ganglion	Air puff, pin-prick and thermal stimulation (radiant heat source). Spontaneous scratching behavior quantified	None – tested in cage (air-puff and pin prick); acrylic rodent restrainer (thermal stimulation)	Ahn et al. (2009a)
Mouse	C57BL/6	Partial ligation	IoN	Von Frey	Animal placed on a mesh floor, covered by a plastic cup	Xu et al. (2008)
Mouse	C57BL/6	Tight ligation	Mental nerve	Von Frey	Animal hand held	Seino et al. (2009)
Mouse	Swiss	CCI	IoN	Thermal (radiant heat source)	Animal hand held	Luiz et al. (2010)
Mouse	C57BL/6	CCI	IoN	Von Frey and Air puff	Loose box restraint	Krzyzanowska et al. (2011)

CCI, constriction injury model; IoN, infraorbital nerve; TG, trigeminal ganglion; LPA, lysophosphatidic acid.

1 Only the earliest reference to the model is indicated.

## Behavioral Testing

The majority of behavioral pain tests currently in use are only applicable to the hindpaws or tail. Thermal tests such as the hotplate/cold plate or hot-water bath immersion are very difficult to perform in the facial region. The commonly used Hargreaves plantar test, which provides a thermal stimulus with the aid of a movable infrared source is a bulky machine – a small adaptor is required for this type of stimulation to be applied in the facial region. Moreover, in order for the heat intensity delivered to be even, the heat source should always be placed at the same distance from the animals' face, which in freely moving animals is virtually impossible.

Mechanical hyperalgesia measurements can be achieved with the Randall Selitto method, which again would be complicated to use in the facial region, or by von Frey hairs. The latter have been shown to be a valuable tool in measuring facial pain responses (Vos et al. 1994).

On the other hand, the specific characteristics of the orofacial region allow for certain functional tests that cannot be performed with other body parts; in particular, gnawing, chewing, and willingness to chew can be observed and quantified. Thus, we can observe food intake decrease following a TMJ inflammation (Harper et al. 2000), reduction in the bite force following masseter muscle injections of CFA (Ro 2005), a decrease in food-pellet-releasing lever pressing and feeding following both TMJ and masseter muscle inflammation (Thut et al. 2007), and decrease in gnawing through objects following similar inflammation (Dolan et al. 2010). All these reflect symptoms in human orofacial pain patients who avoid pain-potentiating chewing. However, in some cases the observable changes in these behaviors may be subtle and it is of interest to also be able to quantify orofacial hyperalgesia in response to a stimulus.

### Pain-related spontaneous behavior

As most of the currently available pain-testing devices prove impossible to use in the facial region, only a relatively small number of studies has been performed to date and most focus on spontaneous responses (see Tables 1 and 2). The most used to date and the most simple, is the formalin test, which involves the injection of the irritant chemical into the upper lip of the rodent and observing the licking and scratching behavior. This model has been first described by Clavelou et al. (1989) and further used by a number of groups in both rats (Luccarini et al. 2004; Raboisson and Dallel 2004) and mice (Luccarini et al. 2006; Bornhof et al. 2011). In TMJ injections of formalin, a head-flinching behavior and chewing-like motions of the mandible were also observed (Roveroni et al. 2001). Formalin is particularly useful for evaluating primary and secondary hyperalgesia alterations in transgenic mice. Capsaicin mustard oil and glutamate are other substances that elicit spontaneous nocifensive behaviors and also have been applied in the orofacial region in rats (Pelissier et al. 2002; Hartwig et al. 2003; Ro and Capra 2006) and mice (Quintans-Junior et al. 2010).

In a study of chronic constriction of the IoN in rats, Vit et al. (2006) measure the “eye-closure response” as an indication of pain, based on the paroxysms of pain in TN. They show that such eye-closure response can be temporarily blocked with an analgesic dose of morphine and demonstrate an analgesic effect of an interfering-RNA directed against Cx43, a protein found in satellite glial cells, thought to be implicated in neuropathic pain. Such method, once sufficiently validated, could be useful for the study of spontaneous neuropathic responses.

Other spontaneous behaviors such as changes in weight, spontaneous grooming, aggression, and other changes in behavior can be monitored in pain studies (Mogil 2009). Vos et al. (1994) have quantified some of such behavior in their seminal article on the chronic constriction of the IoN. They found that animals with the constriction explored less, exhibited freezing like behavior, defecated more, and gained less weight compared with controls. However, such behavioral studies tend to be time consuming and difficult to quantify, and also it is difficult to ascertain whether they indicate stress, pain, paresthesia, or avoidance behavior and most studies performed in orofacial pain do not include measurements of such spontaneous behavior.

The newly developed Rat and Mouse grimace scales, which measure facial “grimaces” of the rodents following a painful stimulus (so far, only used in nonhead areas (Langford et al. 2010; Sotocinal et al. 2011), may prove to be useful in trigeminal pain models. However, it remains to be seen whether the presence of inflammation in the face would affect the quality of the “grimace”. Also, this method is only valuable for pain of short-to-moderate duration and would not be useful for chronic studies.

### Stimulus-evoked behavior testing methods

The whisker pad region of rodents is a tricky area to study stimulus-evoked behavior. This region has a rich mechanosensory receptor sheet, which is stimulated in nearly continuous haptic activities during exploratory behavior, and these complex whisker movements can complicate the testing. On the other hand, the IoN is a large and relatively easily accessible sensory-only nerve, and innervates a large area which has been the region of choice for many studies.

When studying stimulus-evoked behavior in the orofacial region, one of the major pitfalls is the criterion of the “response”. In the paw region, a reflex-like withdrawal of the paw from the stimulation source is usually considered as the response. In the facial region, the responses may vary from scratching and blinking to grimaces and removing the head. All possible responses need to be classified before testing and analyzing. Vos et al. (1994) have set a standard for orofacial pain testing in the first report of IoN-CCI in rats. They have thoroughly studied the rat's behavior following the CCI intervention, including spontaneous activity (face grooming, exploratory behavior) and evoked behavior which included stimulation with various thicknesses of von Frey filaments and a pin prick. Based on the responses, a “response score” was attributed, combining the various criteria. We have recently adapted a simplified version of such quantification in mice, where face-grooming behaviors, withdrawal and aggressiveness toward the probe have been totaled to achieve a response score (Krzyzanowska et al. 2011).

Apart from the challenges of approaching the testing probes to the area of interest, the facial region is tricky to stimulate as rodents tend to actively move their heads, which is especially pronounced in mice. In addition, mice are particularly active and escape when the stimulating object (such as a von Frey hair) is approached. Rats, on the other hand, are much calmer and it is possible to perform stimulations with von Frey hairs, as demonstrated in numerous publications (Vos et al. 1994; Idanpaan-Heikkila and Guilbaud 1999; Deseure et al. 2002; Martin and Avendano 2009; Martin et al. 2010).

In mice, to date, only a few publications have reported the use of von Frey hairs in the orofacial region. Recently, in a study involving partial IoN ligation, the authors have behaviorally tested mice placed on a mesh floor, restricted within a 8-cm-diameter plastic cup from the top, and stimulated by von Frey hairs from underneath (Xu et al. 2008; Aita et al. 2010). With this approach, it can be difficult to see exactly where the filament is stimulating; moreover, the filament advances parallel to the skin rather than at a 90°, as recommended. In this case, it would be impossible to press the filament against the whisker pad region exerting a bend in the filament. In two other studies of mouse neuropathic facial pain, the animal was held by the experimenter during testing with either von Frey filaments (Seino et al. 2009) or a heat source (Luiz et al. 2010). The holding method requires numerous habituation procedures, is stressful for the mouse and results in the animal being held in an unnatural position, restricting its movements, thus limiting the scope of response. In contrast, in studies involving application of an inflammatory agent (carrageenan) to the orofacial area, the mice were allowed to freely move in a steel tank, with the von Frey filaments being applied from above (Yeo et al. 2004, 2008; Vahidy et al. 2006; Poh et al. 2009; Tang et al. 2009). Although relatively unstressful, due to the active nature of the animals it would be challenging to stimulate them and, importantly, it would be difficult to ascertain where exactly the probe touched the face or what response was obtained. We recently proposed an alternative way of restraining the mice, which involves the mouse being placed in a box, with its tail being attached to a special device (Krzyzanowska et al. 2011). Although not entirely stress-free, this set-up allows the animal to move its head and forepaws freely and allows the examiner to observe various types of responses. Also, plasma corticosterone measurements showed this type of set-up to be less stressful than the hand-held method.

While von Frey hairs can be used for determining mechanical thresholds, the air puff method is a useful tool for studying the effect of a completely non-noxious stimulus. Ahn et al. have used this method in several facial neuropathy (Ahn et al. 2009a,b) and inflammation (Ahn et al. 2004; Jung et al. 2006a,b) models in rats to test whether the animals develop mechanical allodynia. They showed that while naïve animals do not respond to an air puff of 40 psi, animals which had an IL-1β induced inflammation or TG compression responded to air puffs of much lower pressure (5 psi). Our group has observed similar results with the air-puff method in mice which underwent an IoN-CCI or CFA inflammation (Krzyzanowska et al. 2011).

Thermal testing of the orofacial region is even more complicated. The machinery needed for the thermal stimulation, such as a tube with the heat beam, is much larger than the von Frey hairs, and approaching such apparatus may scare the animal. Furthermore, the light shining in the animals eye may be unpleasant. The skin of the snout is covered by hair – unlike the paw which has a glabrous surface – which makes it difficult to apply a specific desired temperature. In addition, a thermal probe will first touch the facial hairs and vibrissae, thus activating the low-threshold mechanoreceptors before producing the thermal sensation, thus a radiant heat source is more suitable (Imamura et al. 1997), or the animal should be shaved (Eriksson et al. 2005; Neubert et al. 2005b). The latter situation is not entirely physiological as some of the normal sensory information is transmitted through the facial hair.

In 1978, Rosenfeld et al. designed a facial nociception device which was mounted onto the skull of the animals and delivered heat to the cheek. The responses measured were scratching or face-rubbing by fore or hind limbs. This apparatus, however, requires surgery to install the device and is clearly uncomfortable for the animal and has not been widely adapted. A more practical test, developed by Imamura et al. (1997), involves placing a rat in a restrainer so that only the snout is visible for noxious radiant heat-beam stimulation, at the same time shielding the eyes animals from the heat light. With this apparatus, they showed significant decreases in withdrawal latencies after a constriction of the IoN. In this set-up, the animals had to be thoroughly habituated to the apparatus before behavioral testing in order to avoid any stress-facilitated changes in behavior and analgesia. A similar contraption was reported by Ahn et al. (2009a) who induced neuropathic pain with an injection of the demyelinating agent LPA into the trigeminal ganglion of the rat. They restrained the rats in a cylindrical acrylic restrainer and applied heat stimulus using an infrared thermal stimulator (diode laser) placed 10 cm away from the vibrissal pad. However, they have failed to observe any differences in responses to this stimulus between the vehicle- and LPA-treated groups (Ahn et al. 2009a). This could be due to the nature of the model, which is more sensitive to mechanical stimuli. Other recent studies used infrared irradiation to thermally stimulate the face of mice and rats held by the investigator (Luiz et al. 2010) or of mice restrained in a plastic tube (Shinoda et al. 2011). Both groups, however, do not specify the type of thermal source machine used and the restraint of the animal by the investigator is not optimal (see above). Moreover, Shinoda and colleagues repeatedly anesthetized the animals in order to place them in the plastic tube for the behavioral testing. While behavioral procedures were performed 30 min after anesthesia, one cannot exclude some residual effects of the isofluorane. Several other studies using thermal stimulus have been reported using lightly anesthetized rats (Tzabazis et al. 2005; Niv et al. 2008; Cuellar et al. 2010); however, little more has been published in awake animals.

### Operant behavior paradigms

A new type of mechanical and thermal stimulation has been proposed by the group of Neubert et al. They have developed a set-up which allows for the observation of operant responses to painful stimuli. In this paradigm, the rodent has a choice between receiving a reward (sweet condensed milk) or preventing receiving an aversive (painful) stimulus. In order to receive the reward, the rodent must poke its snout through an opening equipped with a thermode so that the aversive stimulus is obtained at the same time as the reward. The painful stimuli can be heat (Neubert et al. 2005b), cold (Rossi et al. 2006), or a mechanical stimulus (Nolan et al. 2011), resulting in the reduction of the reward-seeking behavior following peripheral inflammation – an observation which has been demonstrated to be reversed with analgesic drugs (Neubert et al. 2005b). This testing system has also been adapted for studies on mice, showing that TRPV1−/− mice are insensitive to the 37–52°C heat range (Neubert et al. 2008).

Another recent study proposes an alternative way of estimating trigeminal pain based on the rodents' natural tendency to gnaw on objects obstructing their passage in a narrow tube (Dolan et al. 2010). They hypothesize that nociception-induced gnawing dysfunction can be used as an index of orofacial nociception in an animal model, reflecting the trigeminal pain-induced unwillingness to chew in humans, and demonstrate this in three different orofacial pain models in mice.

The operant behavior paradigms allow to observe a more spontaneous type of behavior when compared with stimulus-evoked studies. However, they require considerable training and importantly, have a motivational component which makes the interpretation of the pain-related behavior more complicated (Mogil 2009).

## Efficacy of Clinically Used Analgesics in Animal Models of Orofacial Pain

### Clinical approaches

After identification of the orofacial disorder, patients usually receive pharmacological therapy, although in some cases cognitive behavioral therapy and alternative medicine methods are used (Zakrzewska 2010). A correct diagnosis of the syndrome allows for appropriate therapy and improves outcomes. Nevertheless, many orofacial pain conditions remain intractable and a full recovery is often not achieved, even after surgical interventions. Thus, there continues to be a need for new, more effective pharmacological agents.

In inflammatory conditions, such as TMD, the commonly used drugs are nonsteriodal anti-inflammatory drugs (NSAIDs), corticosteroids, tricyclic antidepressants, or benzodiazepines (Table 3; Cascos-Romero et al. 2009; Cairns 2010; Mujakperuo et al. 2010; Zakrzewska 2010). Opioids also can provide effective pain relief to TMD patients, but their use is restricted due to possible opioid dependence (Bouloux 2011). Several systematic reviews have been performed in recent years to evaluate the efficacy of the numerous drugs used in TMD, atypical facial pain, and burning mouth syndrome; however, due to poor standards of the available trials (low numbers, no controls, poor experimental protocol), no clear conclusions could be made as to which drugs are indeed the most effective to treat these disorders (List et al. 2003; Cascos-Romero et al. 2009; Mujakperuo et al. 2010).

**Table 3:** A selection of drugs commonly used in orofacial pain conditions in humans with examples of studies performed in animal models. The table lists examples of both positive and negative studies observed with presented drugs. No attempt was made to critically conclude based on the quality of the studies whether the drug indeed has pain-relieving efficacy. Where possible, reviews have been quoted which have compared various clinical studies and analyzed the drugs efficacy

Type of drug	Specific name	Clinical use/results	Animal pain model^1^	Behavioral test/s	Species	Efficacy/dosage and administration
Local anesthetics	Lidocaine (alphacaine)	TN – effective (Kanai et al. 2006), PHN – effective (Kanai et al. 2010)	Formalin	Face rubbing response observed	Rat	Yes (50 *μ*L upper lip injection) (Dallel et al. 1995)
			IoN-CCI	Von Frey	Rat	Yes (1, 2, and 4% solution; local injection into rostral orbital cavity) (Idanpaan-Heikkila and Guilbaud 1999)
Anti spasticity drugs	Baclofen	TN – effective (Watson 2004; Zakrzewska 2009)	IoN-CCI	Von Frey	Rat	Yes (3, 5, 10 mg/kg, i.p.) (Idanpaan-Heikkila and Guilbaud 1999)
Anticonvulsants (carboxamides)	Carbamazepine	TN – effective, second choice (Watson 2004; Zakrzewska 2009)	IoN-CCI	Von Frey	Rat	Yes (25–50 mg/kg i.p.) No (5–10 mg/kg, i.p.) (Idanpaan-Heikkila and Guilbaud 1999)
			Trigeminal ganglion compression	Air puff		Yes (50 mg/kg i.p.) No (25 mg/kg i.p.) (Ahn et al. 2009b)
	Oxcarbazepine	TN – effective (Zakrzewska 2009)	No animal studies in the trigeminal region						
Anticonvulsants (GABA analogs)	Gabapentin	Poor results in TN (Watson 2004; Zakrzewska 2009)	IoN-CCI	Von Frey	Rat	Yes (100 mg/kg or 2 × 30 mg/kg or 2 × 50 mg/kg i.p.) No (30, 50 mg/kg i.p.) (Christensen et al. 2001)
		MS-TN – some positive reports (Khan 1998; Solaro et al. 1998; Solaro and Messmer 2010) Effective-NP of head and neck (Sist et al. 1997), PHN (Lewis et al. 2007)	IoN-CCI	Air puff, von Frey	Mouse	Yes (2 × 30 mg/kg i.p.) (Krzyzanowska et al. 2011)
			Formalin	Face rubbing response observed	Rat	Yes (ED50 8.27 *µ*g intrathecal) (Grabow and Dougherty 2002)
	Pregabalin	TN and other neuropathic conditions – moderate results (Obermann et al. 2008; Navarro et al. 2011)	No animal studies in the trigeminal region						
Anticonvulsants (benzodiazepines)	Clonazepam	BMS – effective (Zakrzewska et al. 2005)	No animal studies in the trigeminal region						
	Diazepam	TMD – effective (List et al. 2003; Zakrzewska 2010)	No animal studies in the trigeminal region						
Anticonvulsants (others)	Lamotrigine	TN – effective – second choice (Zakrzewska 2009) or add-on (Watson 2004)	IoN-CCI	Von Frey	Rat	No (5–100 mg/kg i.p.) (Christensen et al. 1999)
Tricyclic antidepressants	Amitriptyline	Persistent facial pain and TMD (List et al. 2003; Zakrzewska 2010), PHN (Lewis et al. 2007)	IoN-CCI	Von Frey	Rat	No (0.5, 2, 10 mg/kg) (Idanpaan-Heikkila and Guilbaud 1999)
			Formalin	Face rubbing response observed	Rat	Yes (ED50 value: 14.6 mg/kg s.c.) (Luccarini et al. 2004)
	Clomipramine	TN – unknown efficacy (Zakrzewska 2010)	IoN-CCI	Von Frey	Rat	No (1.5, 6 mg/kg i.p.) (Idanpaan-Heikkila and Guilbaud 1999)
			Partial IoN ligation	Spontaneous face rubbing and rubbing after acetone application	Mouse	Yes (1.5 mg/kg) (Alvarez et al. 2011)
NMDA blockers	Ketamine	TMD – not effective (Castrillon et al. 2008)	Capsaicin	Face grooming response observed	Rat	Yes (0.4, 1.25, 4, 12.5 mg/kg s.c.); improved efficacy when combined with morphine (Alvarez et al. 2003)
Opioids	Morphine	Generally not used for neuropathic orofacial pain conditions in humans; some efficacy in PHN (Lewis et al. 2007)	IoN-CCI	Von Frey	Rat	No (1 mg/kg i.v.) (Idanpaan-Heikkila and Guilbaud 1999)
			IoN-CCI	Von Frey	Rat	Yes (10 mg/kg i.p.) (Deseure et al. 2002)
			Trigeminal ganglion compression	Air puff	Rat	Yes (2 or 5 mg/kg i.p. or 5 mg intracisternal) (Le et al. 2010)
		TMD – intra-articular injections seem to be effective (List et al. 2001)	Mustard oil into TMJ	Jaw muscle response	Rat	Yes (15 nmol intra-articular) (Bakke et al. 1998)
			Formalin	Face rubbing response observed	Rat	Yes (5 mg/kg i.p.) (Clavelou et al. 1989)
			Formalin	Face rubbing response observed	Mouse	Yes (ED50 value: 2.45 mg/kg s.c.) (Luccarini et al. 2006)
			Capsaicin	Face grooming response observed	Rat	Yes (various doses of capsaicin and morphine tested; various administration methods) (Pelissier et al. 2002)
			Capsaicin/glutamate	Face grooming response observed	Mouse	Yes (5 mg/kg i.p.) (Quintans-Junior et al. 2010)
			Carrageenan	Operant behavior paradigm	Rat	Yes (0.5 mg/kg s.c.) (Neubert et al. 2005a)
			CFA	Air puff, von Frey	Mouse	Yes (3 mg/kg i.p.) (Krzyzanowska et al. 2011)
NSAIDs^2^	Acetylsalicylic acid	Commonly used although mostly for acute pain	Formalin	Face rubbing response observed	Rat	Yes (400 mg/kg i.p.) (Clavelou et al. 1989)
	Indomethacin	Widespread use for migraines but little published data can be found for other orofacial disorders	CFA in skin, TMJ, and muscle	Spontaneous behavior and eating habits	Rat	Yes (4 mg/kg i.p.) (Thut et al. 2007)
			Carageenan	Face rubbing response observed	Rat	Yes (2.5 mg/kg) (Rodrigues et al. 2006)
			CFA	Von Frey and thermal stimulation	Rat	Yes (5–10 mg/kg i.p.) (Morgan and Gebhart 2008)
			Trigeminal ganglion compression	Air puff	Rat	Yes (25, 50, 100 *μ*g/10 *μ*L intracisternal) (Yang et al. 2009)
	Dipyrone	Commonly used analgesic although withdrawn from most countries for side effects	Mustard oil in TMJ	Face rubbing response observed	Rat	Yes (19, 57, or 95 mg/kg i.v.) (Bonjardim et al. 2009)
	Diclofenac	TMJ osteoarthritis: effective (Mejersjö and Wenneberg 2008), not effective (Ekberg 1998)	Formalin	Face rubbing response observed	Rat	Yes – second phase only (10 and 30 mg/kg i.p.) (Padi et al. 2006)
	Naproxen	TMJ osteoarthritis: effective (Ta and Dionne 2004)	Formalin	Face rubbing response observed	Rat	Yes (ED50 value: 17 mg/kg i.p.) (Miranda et al. 2009)
Other analgesics	Paracetamol	Commonly used although mostly for acute pain	Formalin	Face rubbing response observed	Rat	Yes (300 mg/kg i.p.) (Clavelou et al. 1989)
			Formalin	Face rubbing response observed	Mouse	Yes (ED50 value: 100.66 mg/kg s.c.) (Luccarini et al. 2006)

TN, trigeminal neuralgia; PHN, postherpetic neuralgia; CCI, constriction injury model; IoN, infraorbital nerve; TMD, temporomandibular disorders; MS, multiple sclerosis; NSAIDs, nonsteroidal antiinflammatory drugs; TMD, temporomandibular joint disorder.

1 In the inflammatory models, the inflammatory substance was injected into the skin unless otherwise indicated.

2 NSAIDs are often prescribed by dentists, general practitioners, and also specialists. However, few conclusive studies in humans have been performed on NSAIDS in TMD and other orofacial pain disorders. For systematic reviews see: Mujakperuo et al. (2010) and List et al. (2003).

### Clinically used drugs tested in orofacial inflammatory pain models in rodents

Morphine and the commonly used analgesic drugs such as paracetamol and aspirin are the drugs of choice in inflammatory pain models in rodents. All three have been shown to be effective in decreasing the face-rubbing behavior following formalin injections in both rats (Clavelou et al. 1989; Eisenberg et al. 1996) and mice (Luccarini et al. 2006). Morphine also has shown to be effective in diminishing face-grooming responses following capsaicin application in rats (Pelissier et al. 2002) and mice (Quintans-Junior et al. 2010) and responses to von Frey hairs and air puff following CFA-induced inflammation in mice (Krzyzanowska et al. 2011). In an operant behavior set-up with a thermal stimulus, Neubert et al. (2005b) have shown morphine to reverse the decrease in heating element contact and condensed milk intake following a carrageenan-induced inflammation. Thut et al. (2007) showed the efficacy of the NSAID indomethacin to prevent the decrease in eating behavior following CFA-induced TMJ inflammation in rat. Other NSAIDs have also been tested and showed efficacy in both rat and mouse models (Table 3; Bonjardim et al. 2009; Miranda et al. 2009). Outside of opioid drugs and NSAIDs, few studies have been performed with other types of drugs in rodent orofacial inflammatory pain models. Amitryptiline (alone or in combination with morphine) and gabapentin both showed efficacy in abolishing the second stage of the formalin response (Grabow and Dougherty 2002; Luccarini et al. 2004) and ketamine reduced capsaicin-induced face-grooming behavior in rats, an effect which was potentiated with morphine (Alvarez et al. 2003). Other authors have tried several noradrenergic antagonists in a rat facial carrageenan model; however, these drugs are generally not the first treatment choice in TMD patients (Rodrigues et al. 2006).

### Clinically used drugs tested in orofacial neuropathic pain models in rodents

The drugs used for the neuropathic conditions vary, depending on the disorder (Table 3). For TN, anticonvulsant drugs such as carmazepine and or baclofen are the first-line treatment options (Watson 2004; Zakrzewska 2009). Lamotrigine, oxcarbazepine, phenytoin, and several other antiepileptic drugs can also be used, sometimes in combination. Gabapentin is a commonly used drug in neuropathic conditions (Moore et al. 2011), however, both Watson and Zakrzewska claim poor results for gabapentin in TN patients (Watson 2004; Zakrzewska 2009). Nevertheless, some reports suggest its efficacy in TN in multiple sclerosis patients (Khan 1998) for neuropathic orofacial pain (Sist et al. 1997) and in postherpetic neuralgia (Alper and Lewis 2002). In postherpetic neuralgia, tricyclic antidepressants seem to have the most efficacy in pain relief, although anticonvulsants, oxycodone and topical creams (capsaicin, lidocaine), eye drops, and nasal sprays (lidocaine; Kanai et al. 2010) are also used (Alper and Lewis 2002; Lewis et al. 2007). As with all drugs, some of the above pharmacological agents are not tolerated well by the patients or none prove to be effective, emphasizing the need for new, alternative medications (Kitt et al. 2000; Watson 2004).

Targeted injections of the local anesthetic alphacaine into the rat rostral orbital cavity resulted in the rapid and transient abolishment of the IoN-CCI induced mechanical hypersensitivity (Idanpaan-Heikkila and Guilbaud 1999) – an observation also mirrored in the clinic as intraopthalmic or intranasal application of local anesthetics has been shown to be advantageous to the patients in many cases (Spaziante et al. 1995; Kanai et al. 2006). The same group has also tested baclofen, carbamazepine, morphine, and the tricyclic antidepressants amitriptyline and clomipramine in the IoN-CCI model and found that only the former was successful in abolishing the allodynic behavior at nonsedative doses (Idanpaan-Heikkila and Guilbaud 1999), although another group reported clomipramine to be antihyperalgesic in a mouse trigeminal neuropathic model at the same low dose that was ineffective in rats (Alvarez et al. 2011). The result for baclofen was confirmed in another study (Deseure et al. 2002). Interestingly, in the above mentioned rat facial neuropathic pain studies, carbamazepine was not effective while it is one of the most commonly used drugs to treat TN in human patients (Rappaport and Devor 1994; Kitt et al. 2000; Watson 2004; Zakrzewska 2009), and several placebo-controlled trials have proven its overall effectiveness (Wiffen et al. 2005). This difference highlights the discrepancies between the IoN-CCI model and the human TN. However, it is important to consider that in the study performed by Idanpaan-Heikkila and Guilbaud, carbamazepine did have an antiallodynic effect at higher doses (25 and 50 mg/kg) which induced motor disturbances and sedation (Ahn et al. 2009b) also found that such high doses of carbamazepine reversed trigeminal ganglion compression-induced pain, but claimed that the motor dysfunction was mild and only present at the initial stages of treatment (up to 90 min) while the analgesic effect was more prolonged (8 h; Ahn et al. 2009b). In human patients, effective doses of this drug are known to induce side effects such as drowsiness and impairment of motor coordination, which correlates with the results of the studies in rats.

Gabapentin is a drug that is often mentioned as one of the drugs to treat neuropathic pain, including that of the head and neck (Sist et al. 1997; Khan 1998; Solaro et al. 1998). However, its effectiveness is disputed in some more recent reports (Watson 2004; Zakrzewska 2009). In rats, Christensen et al. (2001) have shown that the analgesic effects of gabapentin following IoN-CCI depend on the dosing and administration schedule: low single doses of 30–50 mg/kg had no analgesic effect; higher ones (>100 mg/kg) did, but were accompanied by sedation and impaired motor activity. However, splitting the 30 mg/kg doses into several injections resulted in analgesia without undesirable side effects. We have used this dosing schedule in a mouse model of IoN-CCI and found it to be successful in reversing both von Frey hair and air puff–induced allodynia (Krzyzanowska et al. 2011).

Morphine generally has a poor efficacy in TN patients, a result also observed in rats (Idanpaan-Heikkila and Guilbaud 1999). However, a combination of morphine and the NMDA receptor antagonist HA966, which by itself produced no analgesia, has been shown to induce a profound morphine dose-dependent antinociception at nonsedative concentrations (Christensen et al. 1999). These findings have been contradicted by other reports (Deseure et al. 2002) in which a decrease in hyper-responsiveness following treatment with morphine alone was indeed observed, a difference which the authors argue may lie in the method of behavioral testing. More recently, Le and colleagues (2010) have found both i.p. and intracisternal morphine to relieve mechanical allodynia following air-puff stimulation in rats with agar-compressed trigeminal ganglia.

The drug studies in rodents have demonstrated that the inflammatory and neuropathic orofacial models can in some ways be representative of disorders such as TMD and TN and could be used to test new potential treatments. The development of behavioral protocols in mice additionally allows for the study of various genes involved in orofacial pain states with the aid of transgenics. To date, numerous studies have used experimentally induced orofacial inflammation or neuropathy to demonstrate the analgesic properties of a number of novel compounds: the 5HT1A receptor agonist F13640 (Deseure et al. 2002), mitogen-activated protein kinase inhibitors PD98059 and SB203580 (Lim et al. 2007), phospholipase A2 inhibitors (Yeo et al. 2004) – to name but a few.

## Conclusion

It must be taken into account that none of the models described in this review exactly mirror human conditions. For example, human chronic inflammatory pain rarely arises from a peripheral injection of an irritant agent. Also, it is unlikely that clinical cases are caused by a compression of a peripheral branch of the trigeminal nerve such as the IoN or the mental nerve and no animal models exists mimicking human trigeminal root compression by vascular loops. In addition, it is difficult to design animal models for some more complex disorders that are not yet fully understood, such as the burning mouth syndrome.

However, the symptoms observed in the animals after peripheral nerve manipulation such as allodynia to light tactile stimulation, including air currents, are similar to those seen in TN in humans (Kitt et al. 2000; Devor et al. 2002). Inflammatory agents that induce pain in humans also result in nocifensive behavior in orofacial models in rodents and the inflammatory mediators that are upregulated in animals with TMJ inflammation have also been observed in the TMJ synovial fluid of TMD patients (Sessle 2011). These observations, together with the fact that many of the drugs that are effective clinically in TN and TMD also show efficacy in animal models of IoN-CCI or TMJ inflammation, we can conclude them to be valid for testing new possible therapies.

Still, all available models have limitations, in particular those aimed at investigating neuropathic disorders. There is an acute need for more etiology- and pathophysiology-driven models. In the case of TN, models that target the trigeminal root may provide closer resemblance to human conditions. Some new models such as the trigeminal ganglion compression (Ahn et al. 2009b) or demyelination (Ahn et al. 2009a) have taken the right direction and may prove to be useful in mimicking certain human disorders.

Finally, it must be emphasized that only through careful design and interpretation of the behavioral testing could animal modeling be advanced toward a better management of chronic orofacial pain. In general, when studying pain in laboratory animals, whether developing new therapeutic strategies or investigating the mechanisms involved in the pain-generating phenomena, a reliable way of measuring the behavioral outcomes is indispensable. It is important to note that these outcomes depend on a range of variables pertaining to the stimulus-response framework, and that only the former, the physicochemical parameters of the external stimuli, may be reasonably well controlled. However, the many physiological variables involved in transforming the stimulus into a motor response, either as a simple reflex or a complex behavioral performance, are far less controllable (Le Bars et al. 2001). This is why only after precisely defining the pain models and testing conditions, could safe comparisons be made across studies. With this aim, this review has summarized the currently available models of orofacial pain in mice and rats and has provided a critical assessment of the methods used to evaluate behavioral changes following such models.

## Footnotes

1 Apart from a rare condition of “facial migraine” – See Benoliel et al. (2008).
